# Heparan Sulfate Regrowth Profiles Under Laminar Shear Flow Following Enzymatic Degradation

**DOI:** 10.1007/s12195-013-0273-z

**Published:** 2013-02-20

**Authors:** Kristina M. Giantsos-Adams, Andrew Jia-An Koo, Sukhyun Song, Jiro Sakai, Jagadish Sankaran, Jennifer H. Shin, Guillermo Garcia-Cardena, C. Forbes Dewey

**Affiliations:** 1 Department of Mechanical Engineering, Massachusetts Institute of Technology, 77 Massachusetts Avenue, Rm. 3-254, Cambridge, MA 02139 USA; 2 Department of Biological Engineering, Massachusetts Institute of Technology, 77 Massachusetts Avenue, Cambridge, MA USA; 3 Department of Bioengineering, Korean Advanced Institute for Science and Technology, 291 Daehak-ro (373-1 Guseong-dong), Yuseong-gu, Daejeon Korea; 4 National University of Singapore, E4-04-10, 4 Engineering Drive 3, Singapore, Singapore; 5 Department of Pathology, Brigham and Women’s Hospital, Harvard Medical School, 77 Avenue Louis Pasteur, Boston, MA USA; 6 Department of Mechanical Engineering, Massachusetts Institute of Technology, 77 Massachusetts Avenue, Rm. 3-254, Cambridge, MA 02139 USA

**Keywords:** Endothelial glycocalyx, Shear stress, Heparan sulfate, Heparinase, Glycocalyx injury, Transport model

## Abstract

The local hemodynamic shear stress waveforms present in an artery dictate the endothelial cell phenotype. The observed decrease of the apical glycocalyx layer on the endothelium in atheroprone regions of the circulation suggests that the glycocalyx may have a central role in determining atherosclerotic plaque formation. However, the kinetics for the cells’ ability to adapt its glycocalyx to the environment have not been quantitatively resolved. Here we report that the heparan sulfate component of the glycocalyx of HUVECs increases by 1.4-fold following the onset of high shear stress, compared to static cultured cells, with a time constant of 19 h. Cell morphology experiments show that 12 h are required for the cells to elongate, but only after 36 h have the cells reached maximal alignment to the flow vector. Our findings demonstrate that following enzymatic degradation, heparan sulfate is restored to the cell surface within 12 h under flow whereas the time required is 20 h under static conditions. We also propose a model describing the contribution of endocytosis and exocytosis to apical heparan sulfate expression. The change in HS regrowth kinetics from static to high-shear EC phenotype implies a differential in the rate of endocytic and exocytic membrane turnover.

## Introduction

The endothelial glycocalyx layer on the apical surface of vascular endothelial cells is a complex arrangement of proteoglycans, glycosaminoglycans (GAG), and glycolipids that regulate the ability of the endothelium to sense and respond to extracellular physical and chemical cues. The glycocalyx has been implicated in the regulation of athero-resistant gene expression,^10^,^29^ alignment of cells to an imposed flow vector,^44^ modulation of inflammatory pathways,^24^ and modulation of the diffusion of micro- and macromolecules between the vessel lumen and the cell surface.^17^ Structural changes in the glycocalyx are thought to be the initial proatherogenic step that leads to increased affinity of lipoproteins for vascular subendothelium (reviewed in Ballinger *et al.* 6). Moreover, atherosclerotic lesions exhibit heparan sulfate chains with significant oxidative damage, thereby decreasing their ability to bind growth factors.^20^

Heparan sulfate (HS) is the dominant constituent of the glycocalyx. It is a linear sulfated polysaccharide, formed of 40–300 sugar residues, approximately 20–150 nm in length, and is anchored to the apical core proteins, syndecans and glypicans. HS plays an active and necessary role in the cellular response to environmental stimuli.^2^,^11^ It has been implicated in the shear-stress-induced expression of von Willebrand factor and VE-cadherin,^26^ along with the vasoregulatory proteins cyclooxygenase-2 (COX-2) and nitric oxide synthase (eNOS).^26^,^28^ Heparan sulfate also serves as a reservoir for extracellular proteins, such as FGF2, vascular endothelial growth factor, and lipoprotein lipase (reviewed by Sarrazin *et al.* 36), and can modulate the activity of the protein through receptor-ligand interactions.^35^

*In vivo*, HS is cleaved by heparanase, an endoglycosidase capable of selectively cleaving heparan sulfate from the glycocalyx layer while leaving the core protein intact.^9^ Heparanase is found in high concentrations in platelets, macrophages,^41^ and invading tumor cells^42^ and it has been suggested that normal and malignant blood-borne cells may utilize this enzymatic machinery to escape the vasculature.^41^ High levels of glucose and reactive oxygen species, characteristic of diabetes and inflammation, respectively, increase endogenous heparanase production, leading to increased arterial heparan sulfate degradation.^32^ Under shear stress, the enzymatic removal of heparan sulfate from endothelial cells results in their loss of alignment along the flow vector^44^ and attenuates the production of the vasodilator nitric oxide.^15^ Along with matrix metalloproteinase activity, enzymatic degradation of the glycocalyx also provides benign and malignant circulating cells with access to the subendothelial basement membrane.^25^

The glycocalyx provides a force-sensitive region between the flowing blood vessels and the arterial wall whose thickness depends upon the local shear forces imposed by the fluid flowing at its outer surface.^33^ High shear stress increases the rate of glycocalyx biosynthesis on porcine aortic endothelium compared to cells experiencing no shear.^3^ The increase in glycocalyx under high shear stress has been noted elsewhere,^7^,^18^,^39^ but its growth kinetics under shear stress and following heparanase treatment have not been explored until now. Here we report the time course for the regrowth of heparan sulfate following heparinase treatment, and explore the potential influence of endocytosis and exocytosis on the rates of recovery. These findings have application to atherosclerosis,^32^ diabetes,^14^ and cancer disease models,^5^,^14^,^37^ as it is becoming increasingly clear that heparanase expression has wide-reaching and deleterious effects in multiple regions of the vasculature.

## Materials and Methods

### Cell Culture and Reagents

Primary human umbilical vein endothelial cells (HUVEC) were cultured in Medium 199 buffered with 25 mM HEPES and supplemented with 2 mM l-glutamine, 20% fetal bovine serum (FBS, Gibco), and 1% penicillin–streptomycin (Gibco). On the day of use, 50 *μ*g/mL endothelial growth factor (ECGS, Invitrogen) and 100 *μ*g/mL heparin were added. Cells were used passage 1–3 and were kept in a humidified incubator at 37 °C supplied with 5% CO_2_. All reagents were purchased from Sigma unless otherwise specified. Heparinase III (cat. #50-012) was obtained from Ibex (Montreal, Quebec, Canada) and anti-heparan sulfate antibody 10E4 was obtained from US Biological (Swampscott, MA). DyLight 549 IgM secondary antibody was purchased from Jackson Immunoresearch (West Grove, PA). The cell preservation and imaging medium, ProLong Gold, and the nuclear dye, 4′,6-diamidino-2-phenylindole dihydrochloride (DAPI), were obtained from Invitrogen (Carlsbad, CA).

### Heparan Sulfate Removal and Immunostaining

For heparan sulfate removal, the monolayer was exposed to heparinase III (HpaseIII, 15 mU/mL) for 10 min at 37 °C, then rinsed thoroughly with PBS, covered with cell culture medium, and placed in the incubator for a predetermined time interval. At the end of the incubation, cells were fixed with 4% paraformaldehyde for 15 min then blocked with 5% donkey serum for 30 min. Cells were then treated with anti-heparan sulfate antibody (1:100 dilution) for 30 min at 20 °C, rinsed thoroughly, and treated with a fluorescently labeled secondary antibody (1:500 dilution, DyLight 549) for 30 min at 20 °C. Cells were then incubated with DAPI (2 *μ*M for 5 min) and rinsed thoroughly. Cells were quickly dried by aspiration and covered with ProLong gold antifade mounting medium. The mounting agent was allowed to cure for 24 h protected from light before imaging.

### Shear Stress Apparatus

HUVEC were seeded at 2.5 × 10^5^ cells/cm^2^ in Ibidi μ-slide VI flow channels (Ibidi USA, Verona, WI) coated with 0.1% gelatin and allowed to incubate at 37 °C with 5% CO_2_ for 24 h to facilitate attachment and growth to confluent monolayers. Each Ibidi μ-slide had six independent flow channels containing independent HUVEC monolayers. For the duration of shear experiments, the Ibidi chambers were housed in a miniature incubator (Bioscience Tools, San Diego, CA) and temperature was maintained at 37 °C. Each of the six individual channels was attached *via* Luer connectors to a sterile silicone tube that was fed through an Ismatec 32 channel roller pump (Ismatec, Oak Harbor, WA). Medium at 37 °C was flowed over cell monolayers at a rate of 8.5 mL/min, generating a fluid shear stress on the cell surface of 15 dyn/cm^2^.

### Fluorescence Microscopy

Images were acquired using a cooled CCD camera and MaxIm acquisition software (Apogee Instruments) housed on a Nikon Eclipse TE2000U inverted microscope with 20× 0.50 NA PlanFluor objective. Each image represents a 742 × 500 *μ*m field of approximately 300 confluent cells. No fewer than three images were acquired from each channel. Acquired images were stored as 16 bit linear files and processed with ImageJ software. The background fluorescence was defined as the fluorescence observed with cells stained with secondary antibody and DAPI only. Subtraction of the background yielded the final mean intensity values for cell surface heparan sulfate. The reported standard deviation represents the spread of the mean intensity of multiple images, which were acquired over multiple monolayers of the same treatment group. For each experiment involving heparinase, a monolayer of HUVEC was stained for HS. At least three images were acquired and the mean intensity of this group served as the value to which intensity values from all other experimental groups were normalized.

### Cell Shape Evaluation

Phase contrast images obtained at 20 × magnification during heparan sulfate regrowth and immunostaining step were evaluated using ImageJ. Fifteen cells randomly chosen from each image were traced to obtain cell area (*a*) and perimeter (*p*), which were used to calculate the circularity index (*S*), based on the following formula:

$$ S = \frac{{2\sqrt {\pi a} }}{p} $$

The alignment of the cells along the flow axis was calculated by measuring the angle Ø that is created by the intersection of the line connecting the two farthest points at the cell periphery with the flow vector.

### Statistical Analysis

All results are presented as mean ± standard deviation and statistical significance is set to *p* < 0.05. Statistics for normally distributed data, such as from quantitative fluorescence microscopic images, was determined by ANOVA and a Student–Newman–Keuls *post hoc* test if more than 2 groups were being assessed. For circularity index and alignment angle data that are not normally distributed, the Wilcoxon test for unpaired data was used to compare two groups and Kruskal–Wallis test was used to compare data from more than 2 groups. All statistical analyses were performed using Kaleidagraph software (Synergy Software, Reading, PA). Numbers of data points in a set and other information pertinent to a particular data set are given in the figure captions.

## Results

### Heparan Sulfate Levels are Constant Over Time in Static Cells

**Figure 1:**
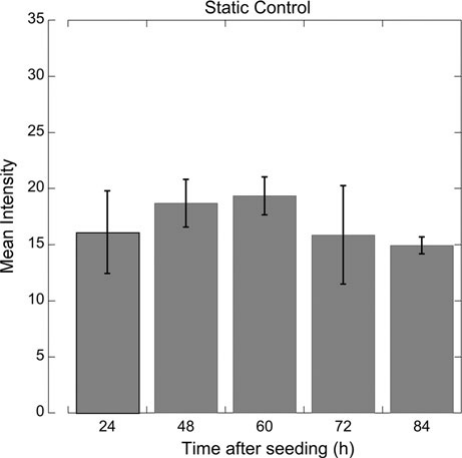
Heparan sulfate signal intensity from apical surface of static cultured HUVEC monolayers is constant up to 84 h after seeding. Grey bars represent the mean heparan sulfate fluorescence intensity of 4–16 monolayer images of different regions taken from 2 to 3 monolayers. No statistical significance between groups (*p* < 0.05)

Heparan sulfate levels did not change significantly for any time between 24 and 84 h after plating (Fig. 1). The cells were seeded at 2.5 × 10^5^ cells/cm^2^, which is a sufficiently high concentration to produce attachment to the substrate within 24 h that does not require further proliferation to achieve confluence. The mean fluorescence intensity values range from 14.9 to 19.3 and no statistical differences were observed between any time points (α = 0.05). The lack of a trend between or significant difference among any of the groups signifies that heparan sulfate content on HUVEC is stable over time and that the acquisition method is valid.

### Heparan Sulfate Levels Increase Under Shear

**Figure 2:**
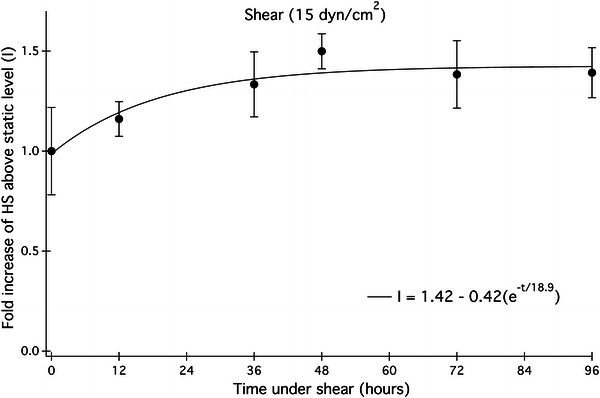
Heparan sulfate growth profile up to 96 h of shear. Data points are mean intensity of anti-heparan sulfate antibody captured by epifluorescence microscopy after discrete time periods of 15 dyn/cm^2^ shear stress. The observed rate constant is 0.053 h^−1^. Error bars are ± standard deviation of 4–16 monolayer images of different regions taken from 2 to 3 monolayers

Application of 15 dyn/cm^2^ laminar shear flow to HUVEC monolayers results in a 40% increase in HS levels as compared to statically cultured HUVEC. The time course for heparan sulfate growth is shown in Fig. 2. HS levels begin to rise as cells are exposed to shear for 12 h and continue to increase toward an asymptote at long times (> 48 h).

To obtain a quantitative measure of the increasing heparan sulfate levels, the data were fitted to the following exponential function:

$$ I(t) = y_{\hbox{max} } - (y_{\hbox{max} } - y_{\hbox{min} } )e^{{\frac{ - t}{\tau }}} $$

This function includes terms for the HS concentration at the plateau (*y*_max_), the starting HS concentration (*y*_min_), and the observed time constant (τ) for the increase in heparan sulfate on the cell surface. The time of half maximum, *t*_1/2_, is the time required for HS abundance to reach a half-maximum on the EC surface by the relationship *t*_1/2_ = τ/ln(2). Values for τ and the time of half maximum (*t*_1/2_) for the curve fit are tabulated in Table 1. The observed time constant, τ is found to be 19 h between 0 and 96 h of shear exposure, indicating that the heparan sulfate concentration on cells has reached its half maximum after 13 h and steady state (which is defined here as within 5% of *y*_max_) after 19 h. The uncertainty in the experiments does not preclude the possibility that the HS abundance continues to increase modestly beyond 96 h.

**Table 1:** Observed time constants (tau) and halflife data (*t*_1/2_) for heparan sulfate growth on endothelial surface

Treatment group	τ^a^ (h)	*t*_1/2_^b^ (h)
15 dyn/cm^2^	18.9 ± 10.2	13.1
15 dyn/cm^2^ + HpaseIII	11.8 ± 3.2	8.2
0 dyn/cm^2^ + HpaseIII	20.2 ± 0.4	13.9

^a^Values are ± fit error

^b^Halflife values are calculated from *t*_1/2_ = τ/ln(2)

**Figure 3:**
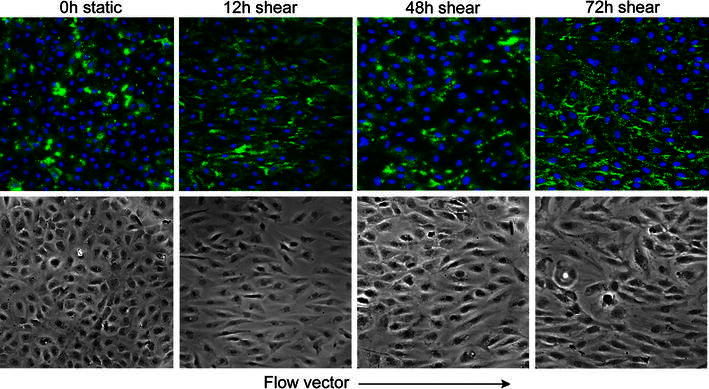
HUVEC monolayer staining for anti-heparan sulfate after 12, 48, and 72 h of laminar flow (15 dyn/cm^2^). Top row, anti-heparan sulfate antibody in green, cell nuclei in blue. Bottom row, corresponding phase contrast image

Representative epifluorescent microscopy images used to quantify HS are presented in Fig. 3. The distribution of HS is heterogeneous between individual cells as well as between multi-cellular areas of the monolayer. The distributions become more homogeneous with exposure to laminar flow (Fig. 3, top row). On cells that have been exposed to shear for 12 h and more, increased heparan sulfate staining is clearly visible.

### Endothelial Cells Align with the Flow Axis

**Figure 4:**
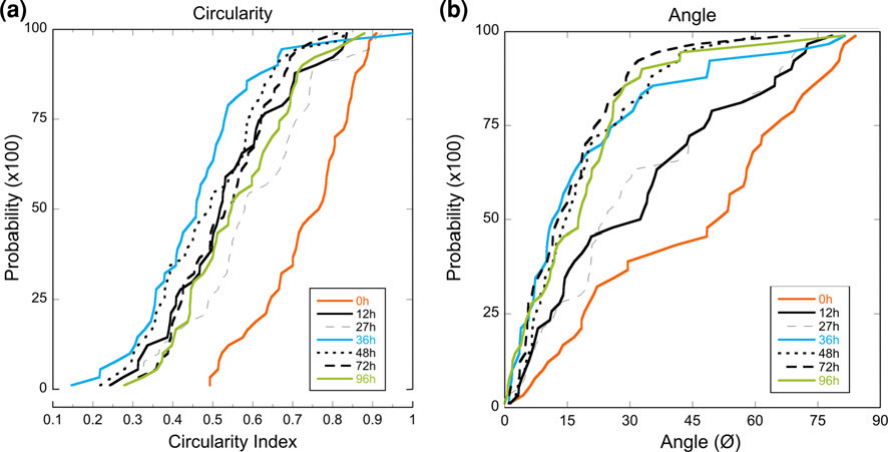
Cumulative probability distributions for cells after time exposed to laminar flow (15 dyn/cm^2^) moving from static (0 h) to 4-day shear (96 h) phenotype. (a) Circularity index decreases significantly for cells after 12 h of shear (*p* < 0.001, *N* = 45) but no further reduction in the circularity index occurs at subsequent time points. (b) Angle of alignment to the flow vector increases significantly after 12 h following heparinase treatment (*p* = 0.01, *N* = 45) and again after 36 h of shear (*p* = 0.005, *N* = 45)

In order to determine the kinetics for cell alignment, phase contrast images were evaluated for changes in circularity index and angle with respect to the flow vector. After 12 h of exposure to laminar flow, we documented that the cells have elongated from the rounded shape characteristic of statically cultured endothelial cells (mean circularity index S_50_ of 0.8) to a significantly lower circularity index of 0.5 (Fig. 4a). There is a significant difference in circularity between statically cultured EC and the 12 h data set (*p* < 0.001), which is not different from time points after 12 h. No statistical differences exist among data sets between 12 and 96 h of laminar shear (*p* = 0.36).

After 12 h of exposure to laminar flow, EC alignment with the flow vector also had significantly increased (*p* < 0.01) from a median angle of 51° to 32° with respect to the flow vector (Fig. 4b). After 36 h, a further significant decrease in the alignment angle is measured (11°, *p* = 0.005). Between 36 and 96 h, no significant difference in angle alignment is detected, indicating that alignment in the direction of the flow vector is complete after 36 h. These data are in agreement with previously reported studies of cellular elongation and alignment^34^,^44^ and form the basis for comparison with cells subjected to heparan sulfate degradation.

### Heparan Sulfate is Restored 12 h After Heparinase Injury Under Laminar Flow

**Figure 5:**
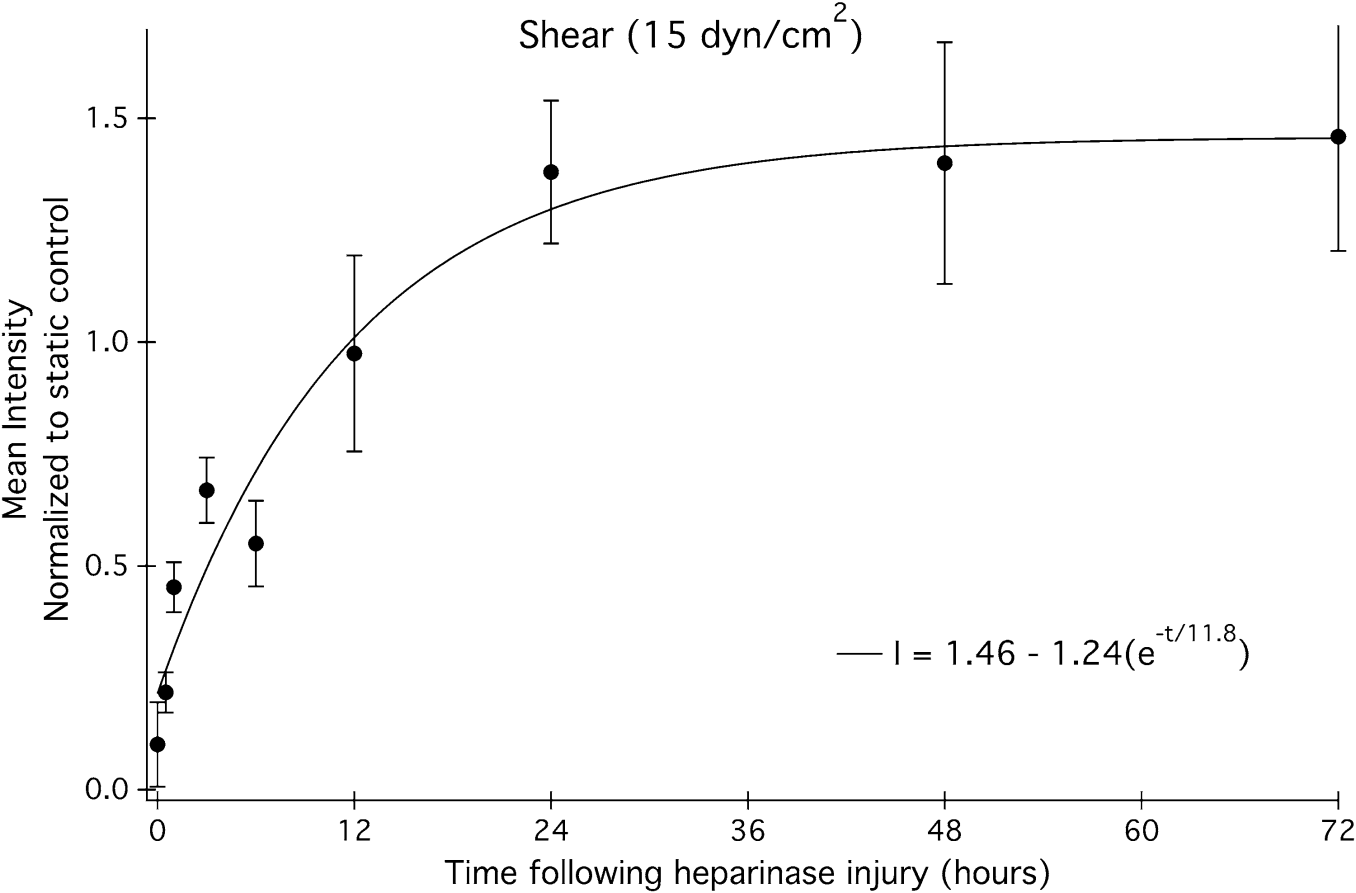
Heparan sulfate regrowth profiles under laminar flow (15 dyn/cm^2^) following heparinase treatment at time 0. Each point represents mean fluorescence intensity of HS from 4 to 9 images. The observed time constant, tau, is 11.8 ± 3.2 h

The HUVEC monolayer was subjected to 24 h of laminar flow at 15 dyn/cm^2^ then briefly exposed to heparinase III (Fig. 5, *t* = 0). Heparan sulfate was allowed to regenerate at 37 °C for discrete time periods under shear and then the relative abundance was measured using quantitative fluorescence microscopy. Data points in Fig. 5 have been normalized to the HS signal from static untreated HUVEC, which were grown in tandem with sheared endothelial cells to serve as a control. The control for the heparanase treatment is Fig. 2, the shear response without heparanase exposure. The control with no shear and no heparanase is a straight line at the unsheared static condition, which is taken as 1.0 for all of the related figures.

The rise in HS levels begins almost immediately; approximately 35% of the starting concentration of HS has returned after 3 h, after which point a plateau emerges where the rate of HS increase slows until after 12 h. After 24 h, the data have reached steady state and no net increase in HS is observed for any time point thereafter. Based on a single exponential model, the observed time constant for the restoration of the heparan sulfate fraction of the glycocalyx is 11.8 h as shown in Fig. 5.

### Heparan Sulfate is Restored Within 20 h Under Static Condition

**Figure 6:**
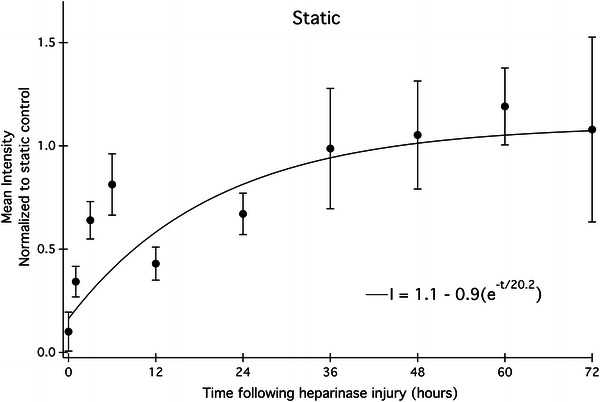
Heparan sulfate regrowth profiles under static conditions following heparinase treatment at time 0. Each point represents mean fluorescence intensity of HS from 4 to 9 images. The observed time constant, tau, is 20.2 ± 0.4 h

**Figure 7:**
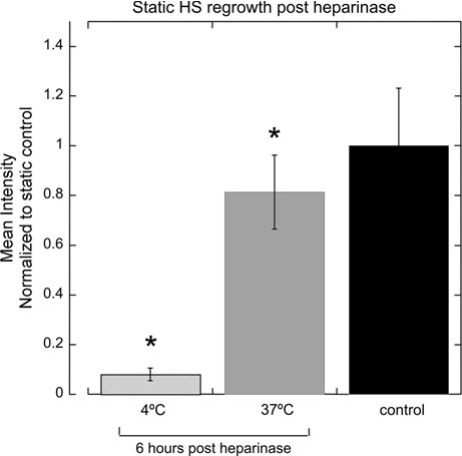
Heparan sulfate regeneration on cell surface is exocytosis-dependent. For 6 h following heparinase exposure, heparan sulfate exocytosis is diminished at 4 °C (shaded bar) and actively restores heparan sulfate at 37 °C (grey bar). Heparinase treatment groups are normalized to the static control condition (black bar) receiving no heparinase. * *p* < 0.05 difference from control. *N* ≥ 4

Experiments analogous to those reported above for cells under shear stress were also conducted on static cultured EC by exposing them to heparinase III. It is found that static cultured EC regenerated heparan sulfate at a slower rate than cells exposed to high shear stress (Fig. 6). Following heparinase exposure, HS exhibited a similar trend as seen in high shear phenotype EC: an initial burst phase of regrowth within the first 6 h, after which time HS levels reach steady state within 20 h. The difference between static and flow conditions lies in the time required to reach a steady state equal to the HS level for each phenotype not exposed to heparinase. Fitting the data to a simple exponential equation yields an observed time constant of 20 h. Thus, the time necessary for HS restoration on statically cultured cells is almost twice as long than the time required for restoration on endothelium under flow. This difference may be attributable to differences in the endocytosis and exocytosis rates for the two phenotypes,

Heparinase-treated endothelial cells in static culture were unable to regenerate apical heparan sulfate after 6 h when the endocytic/exocytotic pathways were inhibited by cold temperature (Fig. 7). Following heparinase injury, cells in static culture incubated for 6 h at 37 °C were able to replenish 80% of the heparan sulfate that is present on untreated control monolayers. Low temperature completely abolishes the energy-dependent exocytosis of nascent heparan sulfate. These data also suggest that the HS turnover on EC follows an energy-dependent mechanism such as endocytosis and exocytosis.

### Cell Alignment Under Shear Stress is Arrested Following Heparinase Treatment

**Figure 8:**
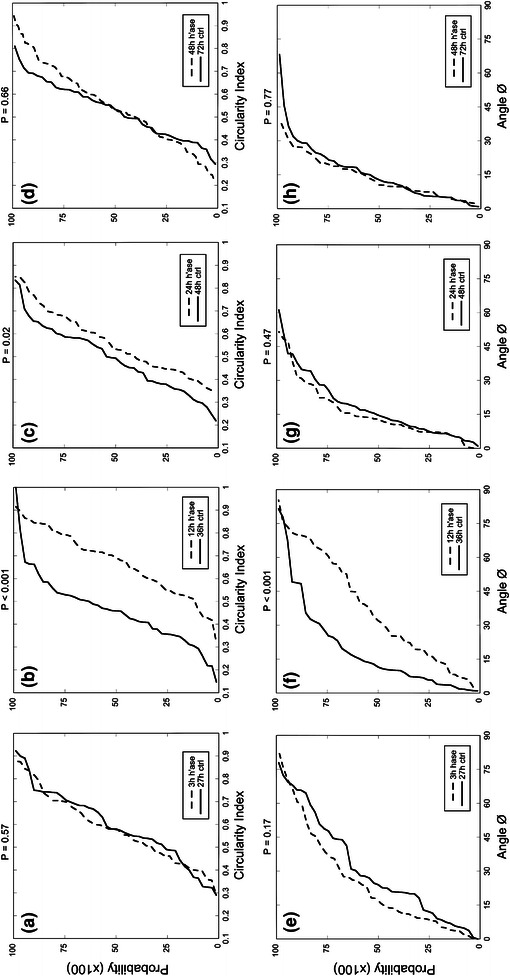
Cumulative probability distributions for heparinase-treated EC under laminar flow (15 dyn/cm^2^) measuring circularity index (a–d) and angle of alignment (e–h). Circularity indices for cells under laminar flow for (a) 3, (b) 12, (c) 24, and (d) 48 h following heparinase treatment and time-matched controls. Significant differences are shown after 12 and 24 h under laminar flow following enzymatic degradation. Angle of alignment data are given for cells under laminar flow for (e) 3, (f) 12, (g) 24, and (h) 48 h following heparinase treatment and time-matched controls. Significant differences are shown at 12 h following heparinase treatment. (*N* = 45 cells per group from 3 separate monolayers)

HUVEC, cultured for 24 h under laminar flow, were briefly exposed to heparinase and were evaluated for changes in cell shape. In Fig. 8, the probability densities are given for the angle of the cell with respect to the flow vector and the circularity index of the cells. No significant difference was observed for either criterion after 3 h under shear following heparinase treatment when compared to control monolayers under laminar flow for the same duration of time (Figs. 8a, 8e). After 12 h following heparinase treatment, there is a significant increase in circularity of the cells when compared to control cells (Fig. 8b) that persists until 24 h following heparinase treatment (Fig. 8c), at which point the circularity has recovered to control values (Fig. 8d). After 12 h following heparinase treatment, the angle of alignment to the flow vector is significantly decreased compared to control (Fig. 8f). By 24 h, the alignment has recovered and there is no difference between heparinase treated EC and control monolayers (Figs. 8g, 8h).

## Discussion

The current results demonstrate that under 15 dyn/cm^2^ of laminar flow, the endothelial heparan sulfate level increases by 1.4-fold after 19 h. The physiological significance of increased glycocalyx under flow has been demonstrated as regions of the carotid artery that are exposed to atheroprotective flow have a significantly thicker glycocalyx than regions under disturbed, or atheroprone, flow.^10^,^39^ In cultured endothelial cells, high shear stress increases production of the gene KLF2,^40^ whose downstream effects include increased production of proteoglycan 1, secretory granule (PRG1) and various sulfotransferases (SULF1, SULF2)^29^ that are present in the Golgi to modify nascent HS. Development of a thicker glycocalyx may serve the atheroprotective phenotype by providing steric hindrances to infiltrating cytokines and macrophages (reviewed in Lipowsky^23^).

Heparan sulfate fluorescence signals, collected at various time points between inception and 72 h following the application of shear stress, were fit to an exponential function that describes the observed cell surface HS as a function of time and the starting and plateau concentrations of cell surface HS (Fig. 2). The time constant for the observed upregulation of HS following shear onset is 18.8 h. If the data were extended past 4 days, the HS levels may continue to rise, as is observed when EC are exposed to shear stress for 7 days and a 2-fold increase in HS is seen.^22^

The rate of the decrease of the circularity index and alignment angle follows the time course for HS upregulation under laminar shear stress (Fig 4). Circularity is significantly decreased, indicating significant cell elongation, after 12 h (Fig 4a) and the angle of alignment to the flow vector has also significantly decreased after 12 h and reaches its maximal alignment after 36 h (Fig 4b). Interestingly, flow conditioned EC exposed to heparinase temporarily increase circularity at 12 h after heparinase treatment (Fig. 8b) and the angle of alignment also temporarily increases by 12 h (Fig. 8f). These results indicate that the loss of the ability of cells to sense shear causes them to become more round and disorganized. In these experiments, the cells are regenerating their HS glycocalyx at the same time the loss of alignment is taking place. Thus, there is a first change towards the static conditioned EC followed by a return to the atheroprotective phenotype following restoration of the glycocalyx (Figs. 8c–8d and 8g–8h). EC without heparan sulfate behave similarly to high shear phenotype EC that are experiencing a sudden loss of shear stress. Remuzzi *et al.* 34 have demonstrated that when flow is removed from high shear cultured EC, the mean elongation of the cells persists for 3–4 h, after which point the decrease in the cell’s aspect ratio is monotonic with time.^34^ In Figs. 8c and 8d, the circularity of the cells has made a significant recovery after 24 h and makes a full recovery after 48 h, indicating that the restoration of HS restores the ability of the cell to sense and respond to an imposed flow vector. The changes in EC alignment are also returned to pre-treatment conditions within 24 h (Figs. 8g, 8h), again reflecting the time scale necessary for static cultured cells to respond to changes in local flow.

Our observations of a glycocalyx with a heparan sulfate moiety that is removed by heparinase and regrows in 1–2 days depending on the flow conditions is contradictory to the report by Potter and Damiano that endothelial cells in culture do not posses any glycocalyx.^31^ In addition to the detection of a robust heparin sulfate layer *in vitro*, we also find that characteristic functions of the endothelial cells to align with the flow direction and change to an elongated form become lost when the glycocalyx HS is removed and the mechanisms supporting these responses are restored when the HS moiety is restored. The apparent absence of a glycocalyx in earlier studies^31^ may have resulted from their method of enzyme delivery. Enzymes were delivered and circulated in medium lacking serum protein, which Adamson and Clough^1^ have shown to collapse the glycocalyx. Also, their *in vitro* model was a small long cylindrical cavity in a collagen gel, and the ability of this model to reproduce other known features of endothelial response reported in previous papers has not been demonstrated. Recent measurements by Ebong *et al.* 12 show significant glycocalyx on *in vitro* endothelial cell cultures. Additional data are now available from confocal microscopy that clearly identify the apical glycocalyx layer *in vitro* and measure HS thickness in excess of 0.5 *μ*m. Furthermore, the specificity of hyaluronidase for hyaluronic acid has not been proven; therefore, its ability to completely remove the glycocalyx, which is composed of a variety of polysaccharides, is a surprising result. Gao and Lipowsky^17^ report that heparanase reduces the volume of the glycocalyx by 60% and hyaluronidase treatment results in a 20% reduction in total glycocalyx volume. The remainder of some glycocalyx would be expected if only certain components are being targeted. We demonstrate that *in vitro* HUVEC can produce a physiologically responsive glycocalyx, as these cells align in the direction of flow, and that a key component of that glycocalyx is restored within a physiologically relevant time frame following acute enzymatic removal.

The overall shapes of the kinetic profiles of both shear-conditioned and static cells recovering from heparinase injury have similar biphasic properties. Within 6–12 h, HS levels have recovered to 60–80% of their final concentration and the rapid increase in HS suggests that the transit time between newly synthesized HS and the Golgi apparatus is fast under both culture conditions. There is evidence to support a quick turnover rate for heparan sulfate. Within 4 h following a pulse-chase experiment, Carter *et al*.^8^ show that only 35% of the initial [^3^H]glucosamine label in the glycocalyx of HMEC-1 endothelial cells remains, suggesting that heparan sulfate is quickly removed from the cell surface and replaced with new material. In Figs. 5 and 6, there is an initial burst phase of heparan sulfate regrowth, after which point the high shear profile rises and plateaus within 12 h; however, the corresponding rate of regrowth under static conditions is much slower. The biphasic nature of the regrowth profiles of both phenotypes may indicate a change in the dominant HS regrowth mechanism after 6 h. This suggests that it is possible that there is a transition in the early injury response from rapid vesicle release from the Golgi to a dominant recycling of endosomes containing unmodified syndecans back to the cell surface.

**Figure 9:**
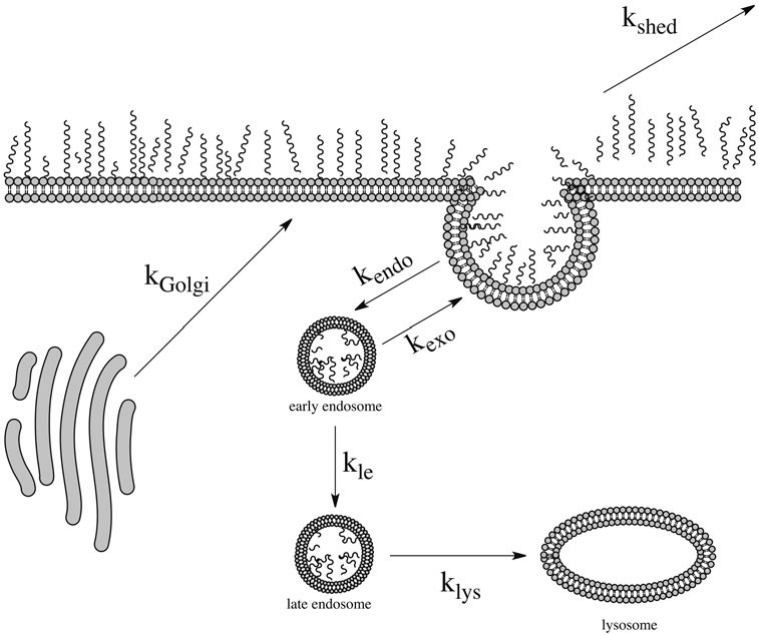
Kinetic model of heparan sulfate turnover from cell surface. Heparan sulfate concentration on cell surface is modeled as a function of the rate of HS production from the Golgi apparatus (*k*_golgi_), endocytosis (*k*_endo_) from the cell surface, and exocytosis from recycling vesicles (*k*_exo_) and from the late endosome (*k*_endole_)

Our proposed model describes the active regeneration and removal of heparan sulfate on the apical surface of endothelial cells and is presented in Fig. 9. Heparan sulfate proteoglycans (HSPG) are produced *de novo* from vesicles that bud from the *trans*-Golgi compartment and appear on the cell surface within 12 min.^38^ Heparan sulfate is then either shed from the surface or internalized into the cell.^4^ Reported rates for internalization vary from 30 min^38^ to 4 h,^43^ but it is generally agreed that the mode of entry is *via* a clathrin- and caveolae-independent mechanism that likely involves macropinocytosis^27^ and/or lipid rafts.^16^,^21^ Following endocytosis, it has been reported that HSPG are swiftly localized to the late endosome,^30^ however, there are reports of a rapid recycling mechanism that may involve early endosomes.^4^,^38^,^45^

Membrane recycling may balance the endocytic removal of surface membrane to maintain cell size and return unmodified proteins and lipids to the surface. Bai *et al*.^4^ have reported syndecan recycling and in kinetic studies, recycling was shown to be a dynamic process that occurs within minutes.^38^ Takeuchi *et al.* 38 have measured syndecan turnover kinetics and calculate that cell surface heparan sulfate recycles to and from an intracellular compartment approximately 10 times before degradation under low calcium conditions. Zimmermann *et al*.^45^ have identified a role for syntenin, which can bind PIP_2_ and syndecan, in the release of syndecans from ADP-ribosylation factor 6 (Arf6)-associated recycling endosomes. A mechanism for heparan sulfate recycling is included in our model as it may be a key component of endothelial homeostasis.

We assume that the concentration of HS in the surface glycocalyx layer is governed by five pathways: (1) uninhibited HS transport of HS-containing vesicles from the Golgi to the membrane surface of the cell; (2) shedding of HS and HS-binding transmembrane proteins from the surface to the plasma; (3) endocytosis; (4) exocytosis; and (5) lysosomal degradation within the cell. Endocytosis and exocytosis are active transport processes which depend, respectively, on the surface concentration and the concentration of HS in vesicles diffusing freely within the cytoplasm. In addition to the three dynamic processes (1), (3), and (4) that are associated with the cell membrane, we include two additional loss mechanisms: (2) shedding of the HS from the surface from circulating cytokine cleavage and (5) the loss of cytoplasmic vesicles through endosomal activity. The model closely resembles the transport processes described by Gross and Lodish for the degradation of erythropoietin.^19^ The following equations describe the change in surface heparan sulfate over time based on these four mechanisms:

1 $$ \frac{{d[{\text{HS}}_{\text{surface}} ]}}{dt} = k_{\text{golgi}} \left[ {{\text{HS}}_{\text{golgi}} } \right] + k_{\text{exo}} \left[ {{\text{HS}}_{\text{ee}} } \right] - k_{\text{endo}} \left[ {{\text{HS}}_{\text{surface}} } \right] - k_{\text{shed}} \left[ {{\text{HS}}_{\text{surface}} } \right] $$

2 $$ \frac{{d[{\text{HS}}_{\text{ee}} ]}}{dt} = k_{\text{endo}} [{\text{HS}}_{\text{surface}} ] - k_{\text{exo}} [{\text{HS}}_{\text{ee}} ] - k_{\text{le}} [{\text{HS}}_{\text{ee}} ] $$

3 $$ \frac{{d[{\text{HS}}_{\text{le}} ]}}{dt} = k_{\text{le}} [{\text{HS}}_{\text{ee}} ] - k_{\text{lys}} [{\text{HS}}_{\text{le}} ] $$

4 $$ \frac{{d[{\text{HS}}_{\text{lys}} ]}}{dt} = k_{\text{lys}} [{\text{HS}}_{\text{le}} ] - k_{\deg } [{\text{HS}}_{\text{lys}} ] $$

**Figure 10:**
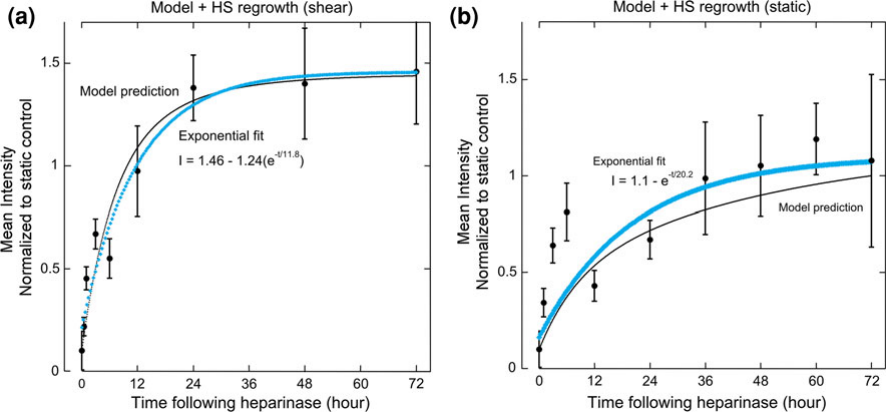
Model predictions closely follow experimental data. Model prediction (thin black line) is plotted with experimental data (filled circles) from heparan sulfate concentration following heparinase treatment for (a) static and (b) shear-conditioned HUVEC (15 dyn/cm^2^). Exponential fit to experimental data is represented by the thick blue line

All concentrations [HS_xx_] are referenced to a unit surface area of the cell, i.e., the cytoplasm is considered well mixed to this operation. Subscript “ee” refers to the cytoplasmic space (early endosomes), and “le” refers to the late endosomes of the terminal lysis pathway. Equation (1) describes the change in cell surface HS as a function of HS production from the Golgi and also replenishment from early endosomes *via* exocytosis, its removal *via* endocytosis and shedding, and its replenishment by exocytosis of early endosomes within the cytoplasm. Equations (2) and (3) track intracellular HS in early and late endosomes, respectively, and Eq. (4) describes the process of transfer from late endosomes to lysosomes and the loss of the degradation products from the lysosomes. The rate constants that are used in the model are listed in Table 2 and Table 3 contains the initial and final concentrations of each compartment described by Eqs. (1)–(4). The model predictions of heparan sulfate regrowth are shown alongside experimental data in Figs. 10a and 10b for the static and shear conditions, respectively.

**Table 2:** Model-derived rate constants

Name	Process	Rate constant (h^−1^)	Static
		Shear	
*k*_Golgi_	*de novo* HS production from Golgi apparatus	0.96	0.96
*k*_exo_	HS exocytosis from recycling vesicles	0.075	0.05
*k*_endo_	HS endocytosis	0.025	0.05
*k*_le_	HS trafficking to late endosome	0.01	0.01
*k*_lys_	HS trafficking to lysosome	0.01	0.01
*k*_shed_	HS cell surface shedding	0.10	0.033
*k*_deg_	HS release from lysosome	0.005	0.005

**Table 3:** Initial and final HS abundances as model parameters

Name	Compartment	Initial concentration^a,b^		Steady state concentration^a^	
		*t* = 0		*t* = 1000 h	
		Shear	Static	Shear	Static
[HS_Golgi_]	Golgi apparatus	0.155	0.05	0.155	0.05
[HS_ee_]	Early endosome	0.4	0.4	0.4	0.4
[HS_le_]	Late endosome	0.4	0.4	0.4	0.4
[HS_lys_]	Lysosome	0.85	1.23	0.85	1.23
[HS_surface_]	Cell surface	0.1	0.1	1.45	1.10

^a^Relative to initial HS surface concentration

^b^Following heparinase treatment

Several assumptions were made in order to develop a sufficiently simple and useful model, as shown in Figure 9. The first assumption is that all labeled heparan sulfate behaves identically. The model for the intracellular behavior of HS was based upon on the shape of the curve that describes [HS_surface_], which was obtained experimentally. The antibodies that targeted cell surface HS did not differentiate between HS on syndecans or glypicans so we do not differentiate between different transmembrane proteoglycans. Second, the rate of HS release from the Golgi is held constant while the rates of membrane vesicles trafficking to the surface is varied, depending on whether the cell has the high shear or static phenotype. Evidence for this behavior comes from Arisaka *et al*.,^3^ wherein high shear stress did not change the synthesis rate of HS but did increase the total rate of transport of synthesized GAG to the cell surface. Last, we assume that HS production is complete as it exits the Golgi, which has been established as the location of HS biosynthesis and nascent enzymatic alteration (reviewed by Esko and Lindahl^13^). We acknowledge that the glycocalyx is a dynamic layer subject to constant real-time modifications in response to environmental changes, however, these details are beyond the scope of the model.

**Figure 11:**
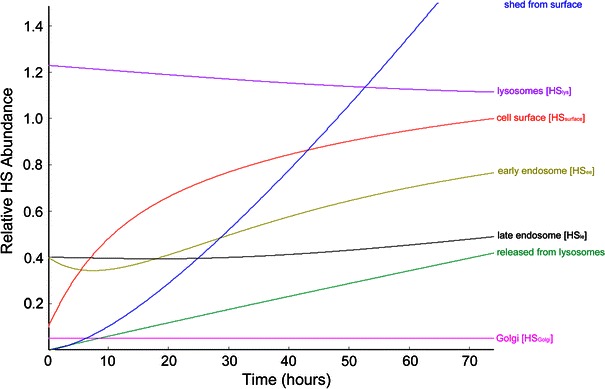
Model prediction for HS trafficking and cell surface regrowth in static phenotype EC following heparinase treatment

The static parameters outlined in Tables 2 and 3 satisfy the conditions set forth by Bai *et al.* 4 that quantifies heparan sulfate at the static endothelial surface, inside the cell, and in the medium. Using pulse-chase experiments with ^35^S radiolabel, Bai *et al*.^4^ determine that the steady-state ratio for HS inside the cell to the surface is 2:1 and similarly reveal that in the untreated wild-type cell, the concentrations of HS on the surface and in the lysosome are approximately equal. While these numbers are not given explicitly in the text, they may be extrapolated by integration of the liquid chromatograms. The ratios derived from Bai *et al.* 4 are mirrored in the concentration of HS in each compartment of the model after steady state is reached (Fig. 11). For the surface concentration, that time is 20.2 h however, for the lysosomal compartment the time to reach steady state is extended because of the time required to bring the upstream early and late endosome compartments to full equilibrium.

**Figure 12:**
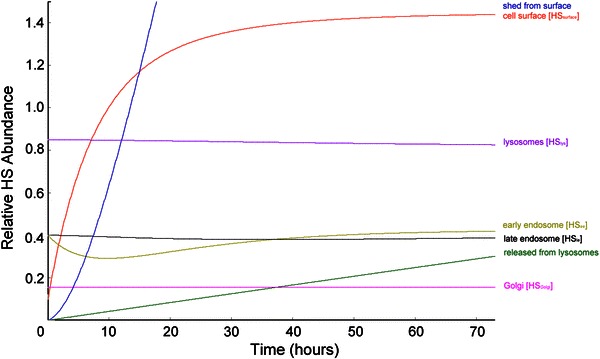
Model prediction for HS trafficking and cell surface regrowth in shear phenotype EC following heparinase treatment

An evaluation of the model for EC under high shear is shown in Fig. 12. To increase the output of HS to the surface, the *de novo* production of HS in the Golgi was increased, while its rate of trafficking to the surface remains unchanged, in agreement with findings from Arisaka *et al.* 3 The plateau abundance of heparan sulfate is reached more quickly in this model due to a 3-fold increase in shedding under shear conditions and a concurrent increase in trafficking of HS from the Golgi to the surface. While it is clear in the literature that shear stress induces shedding under ischemic conditions (reviewed by Mulivor *et al.* 24), the shedding rate has not been explored under high shear stress for otherwise normal endothelium. An increase in the ratio of exocytosis to endocytosis from 1:1 to 3:1 for this model decreases the time necessary to reach steady state on the EC surface. The HS concentration of the intracellular compartment of high shear EC is approximately equal to that of the static EC, which complements the observation by Arisaka *et al.* 3

A close examination of the ability of the model to fit the data for both the static cell recovery and the sheared cell recovery suggests that there may be an additional unmodeled mechanism acting at short times to release HS-containing vesicles to the surface to recover HS much faster than the current model can predict. This would imply that some store of HS-containing vesicles, either in the cytoplasm or in the Golgi themselves, are liberated in the event that the external glycocalyx layer is compromised. It will be an interesting challenge to attempt to identify this unknown HS source that appears in the recovery process.

Shear induced elongation and alignment with the flow vector are attributed in part to the role of heparan sulfate in mechanotransduction.^44^ Here the rate of alignment of the cells to the flow axis is evaluated in tandem with shear-mediated increases in luminal heparan sulfate. Endothelial cells under shear stress begin to align with the flow vector within 12 h, but have not completely aligned until 36 h have passed (Fig. 5b). The circular cell shape that is characteristic of statically cultured endothelial cells has significantly elongated after 12 h, which is on par with significant increases in heparan sulfate as the cells move from static to flow condition. These results indicate that cell motility is a slower event than elongation, but both phenomena reach a plateau after 36 h. Brief removal of heparan sulfate does not have an effect on cell shape or motility, but it is possible that prolonged exposure to enzymatic degradation could cause the cells to revert to a static phenotype with an extended loss of the ability to sense and respond to mechanical stimuli.

## Conclusions

In this work, we demonstrate quantitatively the increases of heparan sulfate on the endothelial surface in response to high shear stress and validate the results using quantitative fluorescence microscopy. We present a time course for the regeneration of heparan sulfate on the endothelial cell surface following heparinase injury and correlate that recovery course with cell elongation mechanics, thus demonstrating an active and relevant glycocalyx present on cultured endothelium. A model for the uptake and release of apical heparan sulfate is presented that couples the experimental HS recovery rate constants with published membrane trafficking constants to provide a phenotype-specific picture of HS trafficking on the endothelial surface.
